# The Application of Porous Carbon Derived from Furfural Residue as the Electrode Material in Supercapacitors

**DOI:** 10.3390/polym16233421

**Published:** 2024-12-05

**Authors:** Zhiyin Zhang, Huimin Hu, Jie Yang, Zhengguang He, Guangyue Zhu, Chang Wen

**Affiliations:** 1 PowerChina HuBei Electric Engineering Co., Ltd., Wuhan 430040, China; zhangzysj@powerchina-hb.com (Z.Z.); wangxy97-huby@powerchina.cn (H.H.); liudhsj@powerchina-hb.com (J.Y.); hezgsj@powerchina-hb.com (Z.H.); 2 Department of New Energy Science and Engineering, School of Energy and Power Engineering, Huazhong University of Science and Technology, Wuhan 430074, China

**Keywords:** biomass waste, porous carbon, supercapacitors, hard template method, heteroatom doped

## Abstract

Resource use is crucial for the sustainable growth of energy and green low-carbon applications since the improper handling of biomass waste would have a detrimental effect on the environment. This paper used nano-ZnO and ammonium persulfate ((NH_4_)_2_S_2_O_8_, APS) as a template agent and heteroatom dopant, respectively. Using a one-step carbonization process in an inert atmosphere, the biomass waste furfural residue (FR) was converted into porous carbon (PC), which was applied to the supercapacitor electrode. The impact of varying APS ratios and carbonization temperatures on the physicochemical properties and electrochemical properties of PC was studied. O, S, and N atoms were evenly distributed in the carbon skeleton, producing abundant heteroatomic functional groups. The sample with the largest specific surface area (SSA, 855.62 m^2^ g^−1^) was made at 900 °C without the addition of APS. With the increase in adding the ratio of APS, the SSA and pore volume of the sample were reduced, owing to the combination of APS and ZnO to form ZnS during the carbonization process, which inhibited the pore generation and activation effect of ZnO and damaged the pore structure of PC. At 0.5 A g^−1^ current density, PC900-1 (FR: ZnO: APS ratio 1:1:1, prepared at 900 °C) exhibited the maximum specific capacitance of 153.03 F g^−1^, whereas it had limited capacitance retention at high current density. PC900-0.1 displayed high specific capacitance (141.32 F g^−1^ at 0.5 A g^−1^), capacitance retention (80.7%), low equivalent series resistance (0.306 Ω), and charge transfer resistance (0.145 Ω) and showed good rate and energy characteristics depending on the synergistic effect of the double layer capacitance and pseudo-capacitance. In conclusion, the prepared FR-derived PC can meet the application of a supercapacitor energy storage field and realize the resource and functional utilization of biomass, which has a good application prospect.

## 1. Introduction

Currently, the energy crises and environmental contamination brought by fossil fuels need to be addressed urgently. Biomass is abundant, easy to obtain, and environmentally friendly, revealing great potential as a renewable energy [1,2]. Biomass waste is an important part of biomass resources, mainly including agricultural waste, forestry waste, industrial waste, and municipal waste. It can potentially pollute the environment, and, thus, its harmless treatment is of great significance. The proper processing and conversion of biomass waste into environmentally friendly carbon materials can realize resource purification and utilization.

Furfural residue (FR) is the industrial biomass waste left after the production of furfural from agricultural and sideline products such as corn cobs, mainly composed of lignin, cellulose, and volatile organic compounds [3]. It is reported that 1 ton of furfural product can produce 10–15 tons of underutilized FR, and the annual production of FR in China reaches 2 to 3 million tons [4]. FR is acidic and will cause serious environmental pollution if it is not properly treated, processed, and utilized [5]. At present, the utilization of FR mainly includes soil culture, biomass fuel, catalytic hydrolysis, and porous materials, among which the conversion of FR into porous carbon has numerous applications. Ao et al. [6] explored the preparation of FR-derived activated carbon using a microwave-assisted system under a CO_2_ atmosphere. The results showed that, at an activation period of 20 min and a microwave activation temperature of 800 °C, the yield, the adsorption capacity of iodine, and methylene blue were 26.8 wt%, 1159.1 mg g^−1^, and 448.0 mg g^−1^, respectively.

Porous carbon shows promising application prospects in energy storage, gas adsorption storage, sewage treatment, catalysis, and other fields owing to its advantages, including a large specific surface area (SSA), large pore volume, controllable porous structure, the ease of modification, and stable physical and chemical properties [7,8,9,10]. A popular technique for producing porous carbon is the hard template approach, while many of the hard templates such as SiO_2_, Fe_2_O_3_, etc., are required to be removed by corrosive acids such as hydrofluoric acid, which may cause environmental pollution [11]. Zinc oxide (ZnO) as a hard template has garnered wide interest because of its low cost, easy synthesis, diverse morphology, and unique physical and chemical properties. When the carbonization temperature is higher than 670 °C, ZnO can react with carbon precursors to produce gas (ZnO + C → Zn + CO) [12], which has an additional pore-forming effect. When the reaction temperature reaches 900 °C, the Zn generated by the reaction tends to evaporate, avoiding the stripping process of pickling or alkali washing, and also contributing to the formation of micropores [13]. For the preparation of porous carbon, ZnO has emerged as a viable hard template. Qin et al. [14] prepared alginate-derived porous carbon by the chemical activation of ZnCl_2_ induced by the nano-ZnO template. Nano-ZnO particles showed triple functions in this process, including the formation of more pores as a hard template, the preparation of zinc alginate hydrogel beads by ZnCl_2_ as a crosslinking agent, and the formation of micropores by ZnCl_2_ as an activator in the process of high temperature carbonization. By covering basic zinc carbonate microspheres with coal tar pitch and coupling with ZnO templates and KOH activation, Jiang et al. [15] constructed porous carbon sheet microspheres. The produced microspheres inherited the template morphology and had a high SSA (up to 2059.43 m^2^ g^−1^).

Porous carbon is frequently utilized in energy storage fields, such as batteries, supercapacitors, and other energy conversion and storage devices, owing to the stable properties and great electrical conductivity [16,17,18,19]. Among them, supercapacitors (SCs) have excellent energy storage efficiency and operational safety, large power density, long cycle life, and fast charge and discharge speed, which have drawn considerable interest in the new generation of energy devices. The abundant pore structure of porous carbon enables it to exhibit high electrochemical properties. Micropores mainly store ions, mesoporous pores are diffusion channels of electrolyte ions, and large pores connect micropores and mesoporous pores as an ion buffer layer, which can shorten the diffusion distance of electrolyte ions [20,21,22]. Based on various energy storage principles, supercapacitors are generally classified into electrical double-layer capacitors and pseudocapacitors, which store and release energy through electrostatic interactions and reversible redox reactions, respectively, while hybrid capacitors are composed of double electric layer capacitors and pseudocapacitors with different energy storage mechanisms [23,24]. Porous carbon, with the advantages of a low price, a diverse pore structure, high conductivity, and good chemical and thermal stability, is a potential electrode material for supercapacitors [25]. Zhang et al. [26] treated the onion with KOH and then pyrolyzed the resulting composite in a N_2_ atmosphere to produce porous carbon derived from the onion, which showed excellent performance in the application of supercapacitors. However, the surface of carbon materials usually has certain hydrophobicity and poor pore accessibility, limiting the electrochemical performance to a certain extent [27]. In general, increasing the SSA of carbon-based materials and introducing heteroatoms can enhance their electrochemical characteristics. An excellent region for the storage and diffusion of electrolyte ions is provided by a large SSA and a suitable distribution of pore sizes, which are important elements in determining the material capacitance and can significantly enhance the electrochemical performance [7,23]. Heteroatom doping can improve the surface wettability of materials effectively, change the distribution of electron clouds around carbon atoms, generate additional active sites in carbon skeleton, and thereby improve the conductivity of carbon materials [28,29].

Therefore, this paper intends to adopt biomass waste FR, ZnO, and ammonium persulfate ((NH_4_)_2_S_2_O_8_, APS) as the carbon precursor, hard template, and nitrogen and sulfur dopants, respectively, to prepare carbon-based materials with a porous structure. The surface morphology, pore structure, elemental composition, and surface functional groups of porous carbon materials were studied. The prepared porous carbon material was applied to the supercapacitor electrode material with high safety and high reliability, and its electrochemical performance in the supercapacitor was evaluated.

## 2. Materials and Methods

### 2.1. Materials

APS (AR), Nano-ZnO (99.9%), HCl (AR), and KOH (95%) were purchased from McLean Biochemical Technology Company (Shanghai, China). Polytetrafluoroethylene dispersion (60 wt%) and conductive carbon black were purchased from Aladdin Reagent Company (Shanghai, China) and Sinopharm Group Chemical Reagent Company (Shanghai, China), respectively. FR was purchased from Rizhao, Shandong Province. The results of the proximate analysis and ultimate analysis of raw FR are shown in Table 1. The volatile content of FR was 70.52 wt%, which contributed to the formation of a rich pore structure during carbonization. The ash content in FR was relatively low, which is 6.43 wt%. The moisture content was only 2.17 wt%, due to the drying treatment of the sample after washing. The results of elemental analysis showed that the contents of C and O are the highest in FR, 50.16 wt% and 41.79 wt%, respectively, and the contents of H, N, and S are low.

### 2.2. Synthesis of FR-Derived Porous Carbon

First, FR and deionized water were evenly mixed and placed in a beaker for 24 h with magnetic stirring to remove soluble impurities and dried at 80 °C for 24 h to remove water. The dried FR was ground in a grinder and screened with a 10-mesh sieve, then sealed, and stored for later use.

As a variable of carbonization temperature, the pretreated FR, nano-ZnO (diameter of 30 ± 10 nm), and APS were mixed at a ratio of 1:1:1, then evenly mixed in a mortar, poured into a porcelain boat, and put into the constant temperature reaction section of the quartz tube in a tubular furnace. After the device was sealed, we passed N_2_ with the purity of 99.9%, checked the air tightness of the device, and purged the air in the reaction tube with N_2_ for a period of time. The samples were heated to the desired temperature (700, 800, 900, and 950 °C) at a rate of 5 °C per minute, and maintained for 2 h. The carbonized sample was washed with HCl (1 mol L^−1^) to remove the remaining ZnO, and the HCl was rinsed with deionized water. Porous carbon was obtained after drying at 105 °C for 12 h.

As the ratio of dopant is variable, the carbonization temperature was set at 900 °C, the ratio of FR, nano-ZnO, and APS was set at 1:1:0, 1:1:0.1, or 1:1:0.5, and other conditions were consistent with the above, to explore the effect of heteroatom dopant APS ratios on the characteristics of porous carbon.

As a control, a sample of FR carbonized at 900 °C with nothing added was prepared, which was named FR900. The samples were named PCT-x. T and x represent the carbonization temperature and the ratio of APS in the sample, respectively. For example, the sample was named PC700-1 as the carbonization temperature was 700 °C, and the ratio of FR, nano-ZnO, and APS was set to 1:1:1.

### 2.3. Sample Characterization

The characterization methods and specific information used for the samples are described in detail in the Supplementary Materials.

### 2.4. Electrochemical Measurement

The detailed description of the electrode sheet preparation process and the particular electrochemical testing techniques adopted are included in the Supplementary Materials.

## 3. Results and Discussion

### 3.1. Physicochemical Properties of Porous Carbon

**Figure 1:**
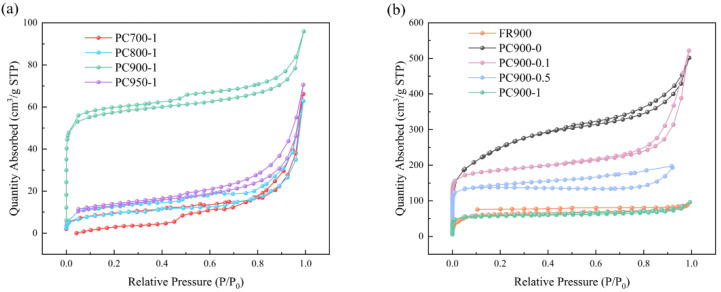
Nitrogen adsorption–desorption isotherms of samples (**a**) at different carbonization temperatures and (**b**) with different APS ratios.

The nitrogen adsorption–desorption isotherms of porous carbon prepared at different processing temperatures and with different APS ratios are shown in Figure 1. PC700-1, PC800-1, and PC950-1 all have obvious hysteresis loops, belonging to type IV curve, while the curves of other samples rise sharply at a lower pressure, belonging to the combination curve of type I and type IV. It shows that both micropores and mesopores exist in the samples prepared at 900 °C. The pore volume, BET surface area, and pore size of all samples are shown in Table 2.

It can be seen from the data that the SSA of PC700-1 and PC800-1 is low and the micropore volume is limited. As the carbonization temperature rises to 900 °C, the SSA of PC900-1 increases to 223.54 m^2^ g^−1^, and the pore volumes also increase significantly. This results from the additional activation of nano-ZnO at higher temperatures to produce micropores, indicating that increasing carbonization temperature in a reasonable range has a positive effect on increasing the SSA of porous carbon. However, when the temperature rises to 950 °C, the SSA of PC950-1 is reduced to 44.33 m^2^ g^−1^, and the volumes of total pore and micropores are also reduced, indicating that the pore structure of porous carbon was destroyed at a higher temperature.

When the proportion of heteroatoms is different, the SSA and total pore volume of the sample without APS are the largest, which are 855.62 m^2^ g^−1^ and 0.648 cm^3^ g^−1^, respectively. The SSA of PC900-1 is much higher than that of FR900, indicating that nano-ZnO plays a role. As the APS ratio rises, both the SSA and pore volume decrease. The SSA is negatively impacted by the addition of APS during the preparation process. This could be because the carbonization process destroys the pore structure of porous carbon through the strong oxidation of APS, which causes the pore structure to collapse and reduces the SSA and total pore volume of prepared porous carbons.

**Figure 2:**
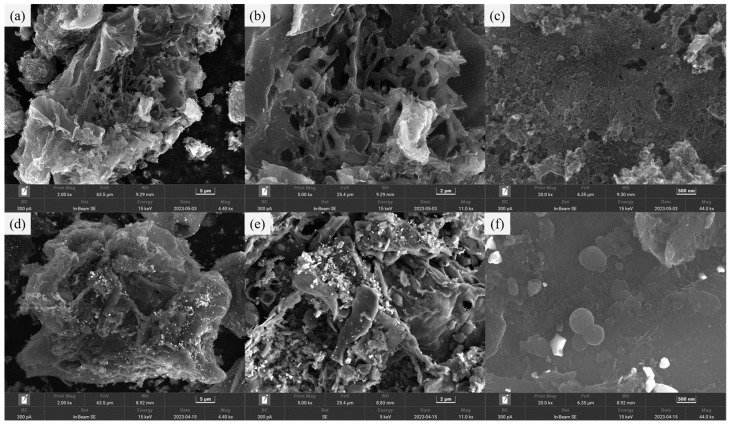
SEM images of (**a**–**c**) PC900-0 and (**d**–**f**) PC900-1.

The scanning electron microscope (SEM) images of PC900-0 and PC900-1 are shown in Figure 2. Both PC900-0 and PC900-1 have interconnected macroporous structures, which is the occupying effect of the ZnO hard template agent [30]. At the same time, there are some tiny pores on the surface of PC900-0 and PC900-1 (Figure 2c,f), which are due to the carbothermal reduction in ZnO at high temperatures and the evaporation of Zn, playing an additional activation role [12]. It can be seen from the image that the pore structure of PC900-1 is not obvious compared with that of PC900-0. Moreover, there are many folds on the surface of PC900-1 (Figure 2e), and the pore structure has an obvious collapse compared with that of PC900-0, speculated to be caused by the strong oxidation and corrosion of APS. The addition of a large amount of APS has a certain destructive effect on the pore structure of porous carbon, which corresponds to the BET results. In addition, PC900-1 has many bright-colored particles on its surface, which may be caused by the addition of APS, for specific reasons discussed below.

**Figure 3:**
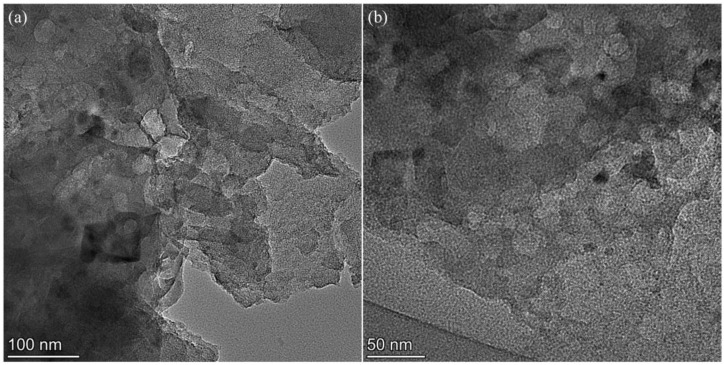
TEM images of PC900-1 (**a**) at scale of 100 nm and (**b**) at scale of 50 nm.

To observe the microscopic pore structure of the sample more clearly, transmission electron microscopy (TEM) images of PC900-1 at different magnifications were obtained (Figure 3). The presence of numerous pores with varying widths in porous carbon is confirmed by Figure 3a. Additionally, mesoporous pores (shown as bright patches in the image) are plainly visible, which promote quick ion transport. Higher magnification TEM images (Figure 3b) show that the carbon skeleton has disordered micropores, which serve as an interface for ion storage.

**Figure 4:**
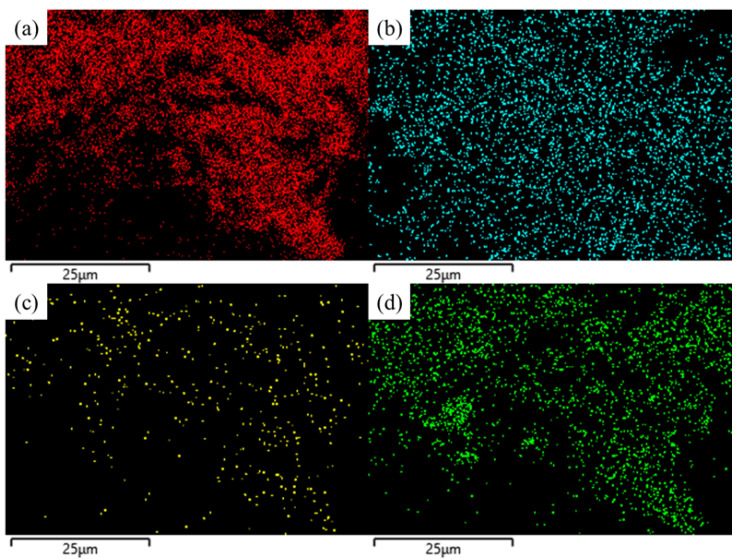
(**a**) C, (**b**) O, (**c**) N, and (**d**) S distribution images of PC900-1.

**Figure 5:**
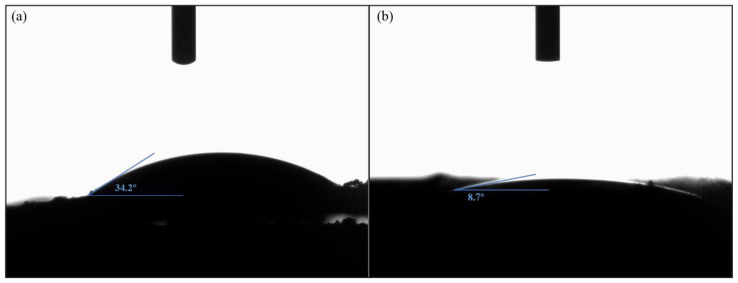
Water contact angle of (**a**) PC900-0 and (**b**) PC900-1.

To evaluate the doping effect of N and S, X-ray energy dispersion spectroscopy (EDS) analysis of PC900-1 was performed, and the EDS test images are presented in Figure 4. C is the most important element contained in PC900-1, and there are O, N and S atoms in the sample, which are evenly distributed in the carbon skeleton. In addition, the distribution of the S element on the carbon skeleton is wider than that of the N element. Homogeneous heteroatom doping is capable of enhancing the reversible Farada y reaction of materials, thereby improving their electrical conductivity and electrochemical properties [31]. Studies have shown that APS can introduce more reactive oxygen species and sulfur functional groups on the porous carbon skeleton [32]. By analyzing the water contact of the samples (Figure 5), the water contact angle of PC900-0 (8.7°) is significantly smaller than that of PC900-1 (34.2°), which fully reveals that heteroatom doping can validly ameliorate the wettability and hydrophilicity of porous carbon, thereby facilitating the diffusion of electrolyte ions and enhancing electrochemical performance.

**Figure 6:**
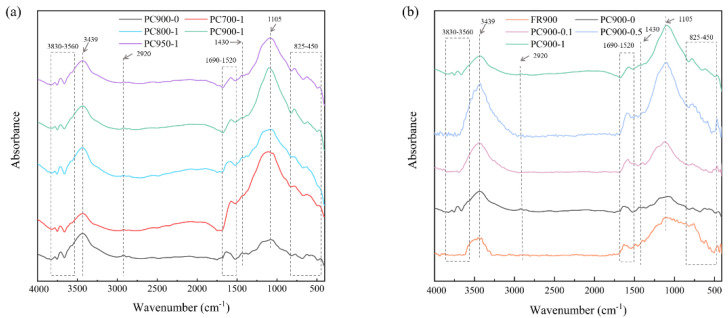
FTIR spectra of different samples prepared with (**a**) different carbonization temperatures and (**b**) different APS ratios.

Fourier transform infrared spectrometer (FTIR) analysis was performed to characterize the chemical functional groups on the material surface, and the spectra are presented in Figure 6. The main characteristic peaks of the samples with the APS addition are similar, and the characteristic absorption peaks are different from those of PC900-0 and FR900. The absorption peak in the range of 3830–3560 cm^−1^ is due to the stretching vibration of O-H [33], and the wide peak appearing around 3439 cm^−1^ is the combined effect of O-H and N-H stretching vibrations [31]. The weak peaks in 2920 and 1430 cm^−1^ correspond to the stretching vibration of –CH_2_ and C=O, respectively. The peaks in the 1690–1520 cm^−1^ range are attributed to the stretching vibration of C-N [9,34] and the stretching vibration of C=C in the aromatic ring [35,36], while the peaks in this range shift slightly in the samples with the addition of APS. It may be the reason that the addition of APS increased the oxygen-containing groups in the samples, and, thus, the asymmetrical stretching vibration of COO- caused the difference [37,38]. The band around 1105 cm^−1^ corresponds to the C-O stretching vibration, C-N vibration, and S=O stretching vibration in the sulfonic acid group [39,40,41]. The peak intensity of the samples adding APS in this band is significantly higher than that of PC900-0, indicating the successful introduction of the sulfur-containing group. The stretching vibration of S=O increases the intensity of the absorption peak, which corresponds to the EDS results. However, it can be seen from Figure 6a that the peak strength of PC700-1 is the strongest, and the peak strength from the risen carbonization temperature is weakened, because the high temperature reduced the content of S=O groups [41]. The absorption peak in the range of 825–450 cm^−1^ indicates the stretching vibration of C-H groups on the carbon surface [37,42].

**Figure 7:**
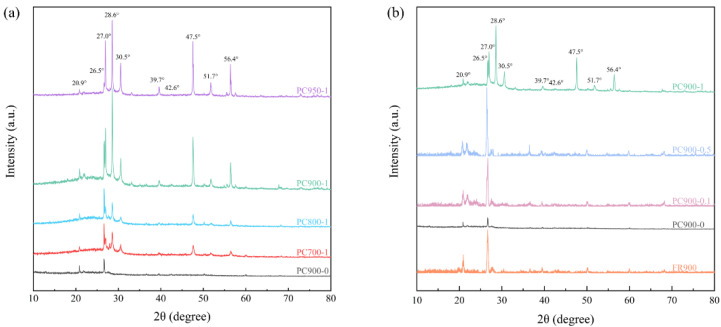
XRD patterns of different samples prepared with (**a**) different carbonization temperatures and (**b**) different APS ratios.

The X-ray diffractometer (XRD) patterns of different samples are shown in Figure 7. Peaks are observed at 2θ = 26.5° and 42.6° for all samples, corresponding to the (002) and (101) crystal faces of graphite, respectively, indicating that all samples are amorphous carbon with a certain degree of graphitization. The (002) peak strength of the samples added with APS is higher than that of PC900-0, which manifests that the degree of graphitization was improved. The peak intensity of PC950-1 at the (002) crystal plane is lower than that of PC900-1, implying that a high carbonization temperature will reduce the degree of graphitization. A diffraction peak was observed at 2θ = 20.9° for all samples, corresponding to the (100) crystal face of SiO_2_, due to the small amount of silicate minerals in the raw FR [6]. Compared with PC900-0 and FR900, diffraction peaks appeared at 2θ = 27.0°, 28.6°, 30.5°, 39.7°, 47.5°, 51.7°, and 56.4° for samples that added APS, which correspond to crystal faces of ZnS on (100), (002), (101), (102), (110), (103), and (112), respectively, due to the reaction of ZnO with APS in the carbonization process, thus producing ZnS. The formed ZnS was not completely removed in the subsequent pickling process and was still doped in the porous carbon, which also explains the bright-colored particles observed in the SEM image of PC900-1. The ZnS crystal face of PC900-1 is more visible than that of samples with a lower APS addition ratio, as shown in Figure 7b, manifesting that more ZnS were generated in PC900-1 as a result of more APS additions. The mass fraction of Zn in PC900-1 was determined by inductively coupled plasma emission spectroscopy (ICP-OES) to be 1.19%, which further confirmed the existence of Zn.

**Figure 8:**
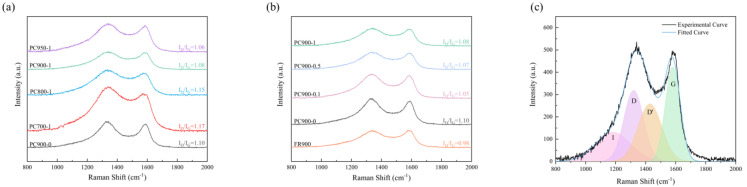
Raman spectra of samples prepared with (**a**) different carbonization temperatures, (**b**) different APS ratios, and (**c**) Raman fitting spectra of PC900-1.

The graphitization degree of the samples was further explored by Raman spectroscopy, and their Raman spectra are presented in Figure 8. Raman spectra can be fitted with four typical bands (Figure 8c, PC900-1 as an example), which are heteroatoms adjacent to carbon atoms (I band at 1182 cm^−1^), defects or disorders in carbon skeleton (D band at 1320 cm^−1^), defects within stacked graphene layers (D’ band at 1428 cm^−1^), and the ordered graphitic sp2-type carbon (G band at 1585 cm^−1^) [43,44]. The intensity ratio of the D and G absorption bands (I_D_/I_G_) is typically utilized to distinguish the extent of defects and disorders in the internal structure of the carbon-based material, namely reflecting the degree of graphitization of samples. As shown in Figure 6a, the I_D_/I_G_ value falls as the temperature rises, suggesting that a higher temperature will result in an increase in the degree of graphitization. FR900 has the lowest I_D_/I_G_ value (Figure 8b), which may be the effect of high temperatures, while further processing will introduce defects or disorders in the carbon skeleton, leading to an increase in the I_D_/I_G_ value. The introduction of APS resulted in the decrease of I_D_/I_G_ value, which gradually increased with the increase in APS addition, illustrating that defects or disorder degrees of the carbon structure increased.

### 3.2. Electrochemical Performance Analysis

**Figure 9:**
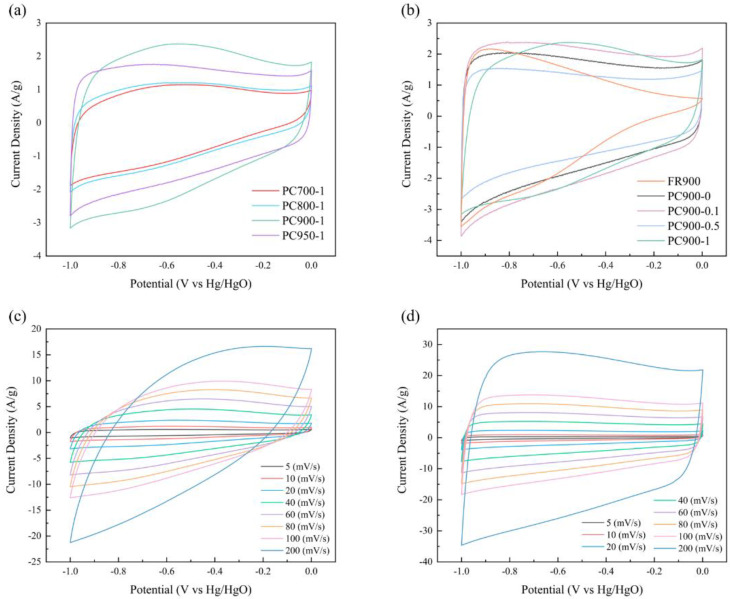
CV curves of samples prepared with (**a**) different carbonization temperatures and (**b**) different APS ratios at a scanning rate of 20 mV s^−1^; CV curves of (**c**) PC900-1 and (**d**) PC900-0.1 at different scanning rates.

The electrode of the supercapacitor was prepared from FR-derived porous carbon, and the standard three-electrode setup in a 6 M KOH solution was used to assess the electrochemical performance. The capacitance characteristics of the electrode prepared from porous carbon were preliminarily evaluated by the cyclic voltammetry (CV) method. Figure 9a shows the CV curves of the samples prepared by different carbonization temperatures at a scanning rate of 20 mV s^−1^. The quasi-rectangular form of the CV curves for all samples indicates a typical double-layer capacitance based on ion exchange and adsorption [31]. It is worth noticing that the CV curve of PC900-1 shows a quasi-rectangular shape with a wide hump, which is the result of the interaction between the pseudo-capacitance provided by the abundant heteroatomic functional groups and the double electric layer capacitance [45]. The above test results prove that the porous carbon material is doped with rich heteroatomic functional groups, which not only contribute to the pseudo-capacitance but also help to improve the conductivity of the carbon skeleton and thus increase the double electric layer capacitance [28]. PC900-1 has the largest integrated area of the CV curve (Figure 6a), exhibiting the highest specific capacitance. Therefore, samples with different APS ratios were prepared at a carbonization temperature of 900 °C, and the impacts of different APS addition ratios on the electrochemical properties of the prepared samples were investigated. As shown in Figure 9b, except for FR900, the CV curves of other samples all show a quasi-rectangular shape, which indicates that FR900 without modification has a poor rate performance. The area of the CV curve of PC900-1 is the largest among samples with different APS proportions, revealing the highest specific capacitance. Figure 9c,d shows that the area of the CV curve increases as the current scanning rate increases, while the CV curve of PC900-1 deforms as the scanning rate increases. The CV curve takes on a spindle shape when the scanning rate hits 200 mV s^−1^, indicating that the PC900-1 electrode has internal resistance. The deviation from the ideal rectangular shape is due to the lack of SSA and the limited pore structure, which increases the electrode resistance and leads to the slow diffusion of electrolyte particles at high scanning rates, despite the abundant heteroatom doping. As the scanning rate rises, the CV curve of the PC900-0.1 electrode material remains rectangular without discernible distortion, showing fast dynamics of the formation of double electric layers and good rate characteristics, owing to its large SSA and developed pore structure (Table 2), which can facilitate the transport of electrolyte ions.

**Figure 10:**
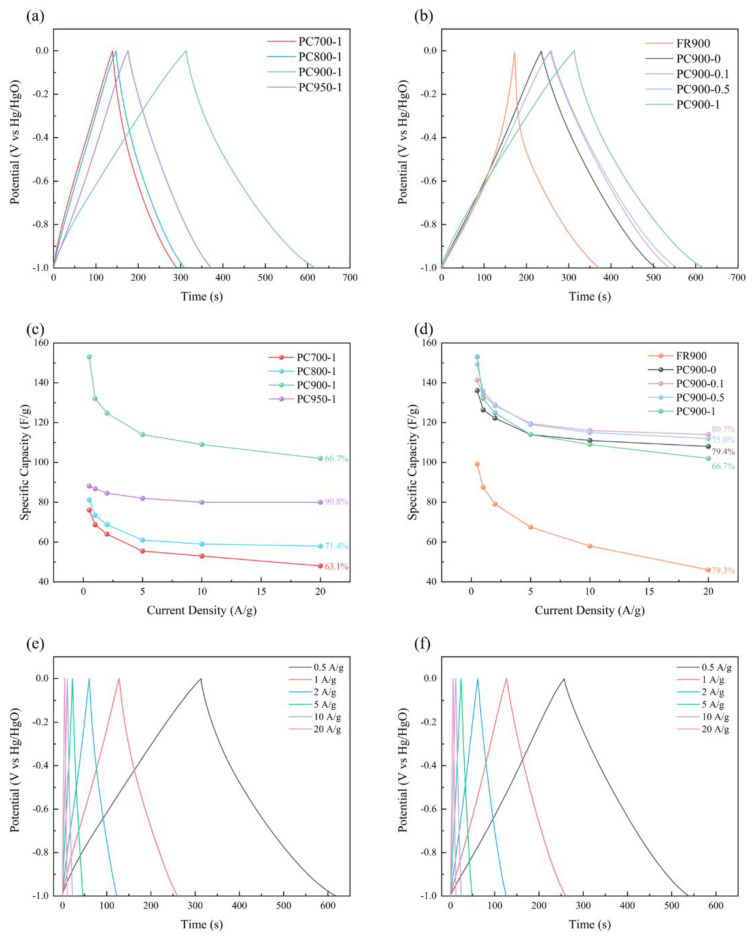
GCD curves of samples prepared with (**a**) different carbonization temperatures and (**b**) different APS ratios at the current density of 0.5 A g^−1^; (**c**) the relationship between the current density and the specific capacitance of samples prepared with different carbonization temperatures and (**d**) different ammonium persulfate ratios; (**e**) PC900-1 and (**f**) PC900-0.1 GCD curves at different current densities.

The electrochemical performance was further examined adopting the constant current charge–discharge (GCD) test. Figure 10a,b displays the GCD charge and discharge curves of various samples at 0.5 A g^−1^ current density, exhibiting regular isosceles triangles, and the curves were slightly curved at the peak value, indicating the pseudo-capacitance characteristics of the electrode material. The GCD curve of FR900 exhibits an irregular triangle, reflecting its poor rate performance, which corresponds to the CV results. In accordance with CV data, PC900-1 has the longest charge and discharge duration of any electrode and the largest specific capacitance at a low current density of 0.5 A g^−1^. The specific capacitance of the sample was calculated from GCD data, and Figure 10c,d shows the relationship between the current density of different samples and the specific capacitance. PC900-1 exhibits the highest specific capacitance of 153.03 F g^−1^ among all samples at 0.5 A g^−1^, revealing good energy storage capacity. However, with the increase in current density, the specific capacitance of PC900-1 drops to 102 F g^−1^ at 20 A g^−1^, and, thus, the capacitance retention is 66.7%, which is caused by its low SSA and limited pore structure. The samples prepared at other carbonization temperatures have lower specific capacitances, because of their low SSA, poor pore structure (Table 2), and limited electron transport capacity. In contrast, PC900-0.1 can maintain a good specific capacitance (114.32 F g^−1^) at 20 A g^−1^, which is higher than that of PC900-1, even though its specific capacitance (141.32 F g^−1^) is lower than that of PC900-1 at 0.5 A g^−1^, and the capacitance retention rate is 80.7%, exhibiting excellent electrochemical stability. Therefore, PC900-1 and PC900-0.1 were selected to plot GCD curves under different current densities, as shown in Figure 10e,f. Under different current densities of 0.5 to 20 A g^−1^, the GCD curve of PC900-0.1 still presents a shape close to a symmetrical triangle. The triangle presented by the GCD curves of PC900-0.1 is more standard than that of PC900-1, benefiting from its suitable pore structure, good rate performance, and reversibility.

**Figure 11:**
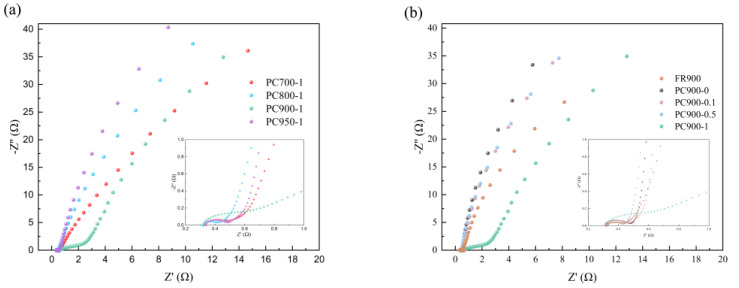
Nyquist diagram of samples prepared with (**a**) different carbonization temperatures and (**b**) different APS ratios.

To investigate the capacitance and resistance characteristics of the electrode, electrochemical impedance spectroscopy (EIS) tests with frequencies ranging from 0.01 Hz to 100 kHz were measured, and the corresponding Nyquist diagrams are shown in Figure 11. All samples present a semicircle shape in the high-frequency region, and the intersection point between the semicircle and the real axis represents its total internal resistance, namely, the value of R_s_ [46]. The diameter of the semicircle is R_ct_, which is related to the process of electrolyte ion transfer between the electrode and the electrolyte. It can be observed that PC900-1 has the largest semicircle diameter and the highest transfer resistance in the high frequency region. The R_s_ and R_ct_ values of different electrode materials fitted by the EIS diagram are summarized in Table 3. PC900-0.1 has a low R_s_ and R_ct_ of 0.306 and 0.145 Ω, respectively, indicating excellent electrical conductivity. However, although PC800-1 also has low R_s_ and R_ct_ values of 0.315 and 0.102 Ω, respectively, it showed limited electrochemical properties from CV and GCD tests due to its poor pore structure. Warburg impedance (Wd) can be obtained from the slope of the curve in the low-frequency region. The larger the slope, the smaller the impedance, which indicates that electrolyte ions have good migration efficiency in the electrode material [31,47]. As can be seen from Figure 8, at low frequencies, the curve slope of PC900-1 is the highest in samples prepared at different carbonization temperatures, while the curve slope of PC900-0.1 is the highest in samples prepared at different APS ratios, indicating a small Warburg impedance, low ion diffusion resistance, and good capacitance performance. However, the curve slope in the low-frequency region in Figure 11 is distinct from the fitted W_d_ value, which indicates that there is a certain error in the fitting data.

**Figure 12:**
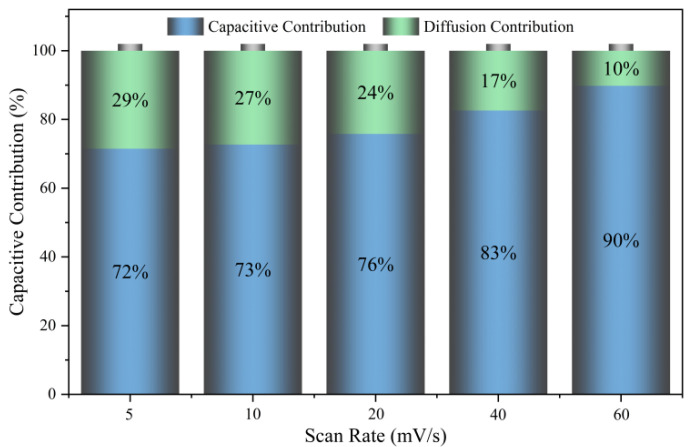
Plots of capacitive and diffusion-controlled contribution at various scan rates of PC900-1.

To investigate the charge storage mechanism of the prepared electrode, the PC900-1 electrode was selected for capacitance contribution analysis, and the contributions generated by the surface capacitance and the diffusion-control process in CV curves at a certain scan rate were quantitatively evaluated. The formula $i=av^{b}$ determines the link between the redox peak current (*i*) and scan rate (*v*) [48]. The charge storage mechanism can be reflected by the b value, and, as the b value is equal to 0.5, it demonstrates a electrochemical process controlled by diffusion, exhibiting battery-like behavior; when b is 1, the current is surface-controlled and exhibits electrochemical behavior similar to that of a supercapacitor, implying a perfect capacitive mechanism [49]. The b value obtained by linear fitting of the oxidation peaks of PC900-1 electrode is 0.938. Figure 12 shows that, as the scan rate rises from 5 mV/s to 60 mV/s, the capacitive contribution rate of the PC900-1 electrode increases from 72% to 90%, demonstrating that the capacitive effect is the main storage mechanism of the electrode.

Table 4 presents a comparison of the specific capacity of various previously reported carbon-based materials prepared with ZnO hard templates or APS doping as supercapacitor electrodes. It is found that the specific capacitors prepared with porous carbon materials in this study are more efficient than other reported materials.

## 4. Conclusions

This study used a simple and feasible method to transform the industrial production waste FR into porous carbon. The morphology and structure characteristics of the prepared porous carbon were analyzed, and the electrochemical properties were investigated by designing experiments and applying it to the electrodes of supercapacitors, thus providing ideas for developing environmentally friendly porous carbon materials. The main conclusions of this study are as follows:

(1) Appropriately increasing processing temperature aids in enhancing the graphitization degree of the produced porous carbon and increasing the SSA, while an excessively high carbonization temperature destroys the pore structure of porous carbon, thus decreasing its SSA.

(2) The addition of heteroatomic agent APS enriched its surface functional groups. The sample PC900-1 has a high sulfur content (19.26 wt%), achieving O, S, and N polyatomic co-doping. However, APS could react with ZnO to form ZnS during carbonization, which adversely affected the activation of ZnO pores and destroyed the pore structure of products. As the APS addition ratio rises, the SSA decreased significantly.

(3) Among all samples, PC900-1 has the highest specific capacitance of 153.03 F g^−1^ at 0.5 A g^−1^, benefiting from the additional pseudo-capacitance provided by numerous surface functional groups. However, the capacitance retention of PC900-1 is only 66.7% at 20 A g^−1^, which is related to the fact that the limited pore structure cannot provide a fast channel for ion transport. Although PC900-0.1 has lower specific capacitance than PC900-1 at low current density, it exhibits a higher capacitance retention rate of 80.7% at 20 A g^−1^, lower equivalent series resistance (0.306 Ω), charge transfer resistance (0.145 Ω), and a CV curve that can remain rectangular at varying scanning rates. PC900-0.1 exhibits good capacitance, charge and discharge stability and rate performance, and excellent electrochemical performance, being an attractive electrode for supercapacitors.

## Figures and Tables

**Table 1:** Proximate analysis and ultimate analysis of furfural residue.

Methods	Proximate Analysis (wt%, ad)				Ultimate Analysis (wt%, daf)				
	Moisture	Ash	Volatile	Fixed Carbon	C	H	N	S	O
furfural residue	2.17	6.43	70.52	20.88	50.16	5.37	2.13	0.55	41.79

ad: air dried basis; daf: dry ash-free basis.

**Table 2:** Pore structure parameters of the samples.

Sample	S_BET_ (m^2^ g^−1^)	V_total_ (cm^3^ g^−1^)	V_micro_ (cm^3^ g^−1^)	D (nm)
FR900	229.15	0.138	0.0601	5.55
PC700-1	25.98	0.050	0.0017	13.75
PC800-1	24.31	0.045	0.0024	13.34
PC900-0	855.62	0.648	0.2365	6.56
PC900-0.1	707.71	0.563	0.2109	14.45
PC900-0.5	557.71	0.341	0.2079	12.41
PC900-1	223.54	0.117	0.0701	8.21
PC950-1	44.33	0.063	0.0052	15.54

**Table 3:** Proximate analysis and ultimate analysis of furfural residue.

Sample	R_s_ (Ω)	R_ct_ (Ω)	W_d_ (Ω)
FR900	0.318	0.119	1.67
PC700-1	0.336	0.143	0.484
PC800-1	0.315	0.102	0.985
PC900-1	0.313	0.358	0.152
PC950-1	0.334	0.141	1.163
PC900-0	0.309	0.165	2.450
PC900-0.1	0.306	0.145	1.157
PC900-0.5	0.331	0.096	2.012

**Table 4:** Comparison table of specific capacitance in similar studies.

Electrode Materials	Electrolyte	Specific Capacity (F g^−1^)	Current Density (A g^−1^)	Ref.
sucrose-derived porous carbon by ZnO template	1 M TEABF_4_/acetonitrile	130	1	[50]
sponge waste-derived porous carbon with ZnO	1M Na_2_SO_4_	133	0.2	[51]
coal tar-pitch-derived porous carbon with ZnO	6 M KOH	172	0.1	[52]
resorcinol–formaldehyde by ZnO template	0.5 M Na_2_SO_4_	51.8	0.5	[53]
poly(3,4-ethylenedioxythiophene) by ZnO template	0.5 M Na_2_SO_4_	89	0.5	[54]
FR-derived porous carbon by ZnO template	6 M KOH	153	0.5	This study

## Data Availability

The original contributions presented in this study are included in the article/Supplementary Material. Further inquiries can be directed to the corresponding authors.

## Supplementary Materials

The following supporting information can be downloaded at https://www.mdpi.com/article/10.3390/polym16233421/s1.
