# Supplementary material for: The Application of Porous Carbon Derived from Furfural Residue as the Electrode Material in Supercapacitors
Source: Polymers (Basel). 2024 Dec 5;16(23):3421. doi: 10.3390/polym16233421 (PMC11644338; doi:10.3390/polym16233421)
Supplement: Supplementary file 1 [file polymers-16-03421-s001.zip › polymers-3313966-supplementary.pdf]

Supplementary Material

The application of porous carbon derived from furfural residue as the electrode material in supercapacitor

Zhiyin Zhang [1], Huimin Hu [1], Jie Yang [1], Zhengguang He [1], Guangyue Zhu [2*] and Chang Wen [2*]

[1] PowerChina HuBei electric engineering Co.,LTD, Wuhan 430040, China. zhangzysj@powerchina-hb.com (Z.Z.); wangxy97-huby@powerchina.cn (H.H.); liudhsj@powerchina-hb.com (J.Y.); hezgsj@powerchina-hb.com (Z.H.)

[2] Department of New Energy Science and Engineering, School of Energy and Power Engineering, Huazhong University of Science and Technology, Wuhan 430074, China; zhugy@hust.edu.cn (G.Z.); wenchang@hust.edu.cn (C.W.)

[*] Correspondence: wenchang@hust.edu.cn; Tel: 86-27-87544779

1. Materials and methods

1.1. Sample characterization

The specific surface area (SSA) and pore structure parameters of the samples were obtained by using ASAP 2460 SSA and pore diameter tester under the condition of liquid nitrogen temperature of 77K. The BET and t-plot methods were used to determine the SSA and micropore area. The surface morphology and structure of the porous carbon were analyzed by TESCAN MIRA LMS scanning electron microscope (SEM) of Czech Republic, and X-ray energy dispersion spectroscopy (EDS) were carried out on OxfordX-MAX type from UK to observe the distribution of typical elements C, N, O, and S. The images of sample by transmission electron microscopy (TEM) were obtained employing FEI Titan G2 60–300 electron microscope, Japan. The percentage of Zn in the sample was determined using inductively coupled plasma emission spectroscopy (ICP-OES) via Agilent 700, America. The water contact angles were measured through Dataphysics Contact Angle System OCA20. The US Thermo Scientific Nicolet iS20 Fourier transform infrared spectrometer (FTIR) was used to determine the surface functional groups of the samples, and the test resolution was 4 cm^{-1} and the wave number ranges from 400 to 4000 cm^{-1}. The crystal structure of the samples was analyzed by Bruker D8 Advance X-ray diffractometer (XRD) produced in Germany, with a scanning Angle range of 10-80° and a scanning speed of 5° min^{-1}. Raman spectra were carried out to further evaluate the crystalline structure of the samples by LabRAMHR-800 (France) at 532 nm.

1.2. Electrochemical measurement

Porous carbon, conductive carbon black, and PTFE were mixed at a ratio of 8:1:1, then anhydrous ethanol was added and stirred in an agate mortar until the slurry became smooth and uniform. Carbon sheets with a thickness of about 100 μm were prepared by a roller press and cut into 1×1 cm rectangular squares. Working electrodes were obtained by vacuum drying at 80 ℃ for 12 hours. The electrode sheet was pressed into a 1×2 cm rectangular nickel foam fluid collector by a tablet press, and the complete electrode material was obtained. The quality of the electrode sheet was determined by the difference in the quality of the nickel foam before and after the wafer pressing.

In the three-electrode system test, the test was carried out in a standard 6 M KOH electrolyte, with the prepared electrode, the platinum electrode, and the Hg/HgO as working electrode (WE), opposing electrode (CE), and reference electrode (RE), respectively. First, the produced electrode sheet was moistened in the electrolyte, and 50 cycle scans were carried out with a sweep speed of 50 mV per second. After the test system was stabilized, cyclic voltammetry (CV), constant current charge-discharge (GCD), and electrochemical impedance spectroscopy (EIS) were conducted on an electrochemical workstation. The working potential of the electrolytes was -1.0 V to 0 V for CV and GCD tests. EIS were carried out at open circuit potential with an amplitude of 5 mV in the frequency range of 0.01 to 10^5 Hz. Based on the GCD data, Eq. (1) was used to determine the electrode material specific capacitance (C, F g^{-1}).

$$C = \frac{I\Delta t}{m\Delta V} \quad (1)$$

Where, I is the loading current (A), Δt represents the discharge time (s), m is the mass of

the active substance on the electrode (g), and ΔV is the discharge point voltage (V).
